# Predictability modulates neural response to eye contact in ASD

**DOI:** 10.1186/s13229-022-00519-0

**Published:** 2022-10-29

**Authors:** Adam J. Naples, Jennifer H. Foss-Feig, Julie M. Wolf, Vinod H. Srihari, James C. McPartland

**Affiliations:** 1 Child Study Center, Yale University School of Medicine, New Haven, CT USA; 2 Department of Psychiatry, Mount Sinai Icahn School of Medicine, New York, NY USA; 3 Seaver Autism Center for Research and Treatment Mount Sinai Icahn School of Medicine, New York, NY USA; 4 Department of Psychiatry, Yale University School of Medicine, New Haven, CT USA; 5 Center for Brain and Mind Health, Yale University School of Medicine, New Haven, CT USA

**Keywords:** N170, P300, Autism, Eye tracking, ERP, Social neuroscience

## Abstract

**Background:**

Deficits in establishing and maintaining eye-contact are early and persistent vulnerabilities of autism spectrum disorder (ASD), and the neural bases of these deficits remain elusive. A promising hypothesis is that social features of autism may reflect difficulties in making predictions about the social world under conditions of uncertainty. However, no research in ASD has examined how predictability impacts the neural processing of eye-contact in naturalistic interpersonal interactions.

**Method:**

We used eye tracking to facilitate an interactive social simulation wherein onscreen faces would establish eye-contact when the participant looked at them. In Experiment One, receipt of eye-contact was unpredictable; in Experiment Two, receipt of eye-contact was predictable. Neural response to eye-contact was measured via the N170 and P300 event-related potentials (ERPs). Experiment One included 23 ASD and 46 typically developing (TD) adult participants. Experiment Two included 25 ASD and 43 TD adult participants.

**Results:**

When receipt of eye-contact was unpredictable, individuals with ASD showed increased N170 and increased, but non-specific, P300 responses. The magnitude of the N170 responses correlated with measures of sensory and anxiety symptomology, such that increased response to eye-contact was associated with increased symptomology. However, when receipt of eye-contact was predictable, individuals with ASD, relative to controls, exhibited slower N170s and no differences in the amplitude of N170 or P300.

**Limitations:**

Our ASD sample was composed of adults with IQ > 70 and included only four autistic women. Thus, further research is needed to evaluate how these results generalize across the spectrum of age, sex, and cognitive ability. Additionally, as analyses were exploratory, some findings failed to survive false-discovery rate adjustment.

**Conclusions:**

Neural response to eye-contact in ASD ranged from attenuated to hypersensitive depending on the predictability of the social context. These findings suggest that the vulnerabilities in eye-contact during social interactions in ASD may arise from differences in anticipation and expectation of eye-contact in addition to the perception of gaze alone.

**Supplementary Information:**

The online version contains supplementary material available at 10.1186/s13229-022-00519-0.

## Introduction

Autism spectrum disorder (ASD) is a neurodevelopmental disorder characterized by preference for routine, motor stereotypies, sensory sensitivities, and pervasive difficulties with social communication. One of the earliest occurring and most impactful social symptoms of ASD is difficulty establishing and maintaining eye-contact and interpreting gaze-related cues [1–7]. Direct eye-contact has been reported as distracting or anxiety-inducing by many people with ASD [8, 9], and teaching people to use gaze effectively in social interactions is a common component of many interventions [10]. However, while disruptions in eye-contact occur frequently in natural social interactions and in clinical observation, people with ASD often display normative performance maintaining, detecting, and following gaze in structured interventions or experiments with explicit instructions [11–13]. This discrepancy hinders understanding of the processes underlying atypical eye-contact and gaze processing in ASD, creating an obstacle to the development of more effective treatments and diagnostic tools.

The discrepancy between experimental findings and clinical observations may relate to the interplay between bottom-up stimulus-driven features and top-down context and task demands. Research in humans and animals indicates that direct eye-contact is processed differently than other visual stimuli. Direct gaze increases arousal as measured by skin conductance [14], is detected more rapidly than averted gaze [15, 16], and is processed by specialized brain regions [17], such as the superior temporal sulcus (STS). However, despite the privileged status of eye-contact, contextual factors, such as task demands, modulate these effects 18]. Detection of direct gaze can be attenuated in visual search tasks with explicit instruction; judgments of gaze direction can be biased by prior experience, face orientation, or emotional expressions; and brain activity to eye-contact can be modulated via explicit instruction or implicit expectations [19–21]. In experimental or interventional settings, when task demands are explicit, participants can guide and modulate their attention accordingly, e.g., to detect images of faces displaying eye-contact or to follow someone’s gaze when instructed to do so. However, in natural interactions, situational demands are implicit and imperfectly predictable, and there may be no explicit cues to prioritize attention to faces or gaze. A failure to flexibly adapt to social context in this way may account for compromised perception of eye-contact and use of gaze in social interactions in ASD.

Indeed, challenges in flexibly adapting to changing contexts, both explicit and implicit, are well-documented in ASD, are part of the diagnostic criteria [22], and are increasingly thought to emerge from differences in how people with ASD generate predictions and expectations about the world around them [23–26]. There is a growing body of evidence that these challenges extend to the most basic levels of sensory perception, such that people with ASD are less effective in using top-down information to modulate low-level sensory processing [27–30]. This atypical modulation is reported across multiple sensory modalities and is thought to underlie sensory hyper- and hypo-sensitivities with cascading effects that lead to symptoms across multiple domains, including attention, anxiety, and social function [24]. In this way, atypical modulation of perception could derail successful social interactions in ASD in two ways: (1) In the moment-to-moment cadence of a social interaction, the perception of gaze might be out of step with a social partner, disrupting synchrony of the interaction; (2) over a lifetime of such disrupted social interactions, eye-contact and direct gaze may become distracting or anxiety-inducing rather than an informative social signal.

Cognitive neuroscience methods offer sensitive tools to investigate the perception of eye-contact and its modulation. Since instances of shared gaze between people frequently last less than a second [31], neural processes supporting flexible gaze processing must operate on commensurate time scales. The electroencephalogram (EEG) records brain activity at this pace, and two event-related potentials (ERPs), the N170 and P300, index facets of gaze perception occurring in less than a second. The N170 is an ERP occurring approximately 170 ms after the onset of a visual stimulus that is measured over right occipital scalp and displays larger amplitudes and earlier latencies to faces and eyes relative to other stimuli [32]. The N170 is reliable within people over time and is associated with performance on standardized measures of face memory [33]. Changes in N170 amplitude reflect variation in strength of neural activation, and changes in latency index neural efficiency. The P300 is a positive deflection measured over central scalp that displays larger amplitudes to stimuli that are motivationally relevant, e.g., stimuli targeted for behavioral response [34, 35]. Importantly, even in the absence of an explicit task, social stimuli such as faces and eyes evoke robust P300 responses, suggesting that such stimuli automatically engage the preparation of a response by virtue of their social significance [34]. Both the N170 and P300 show reliable top-down modulation in response to social context. N170 amplitude is influenced by contexts including facial feature (eye vs. mouth movement) [36], facial realism (real vs. photographed) [37], sequence of stimulus presentation (faces preceded by non-face vs. face stimuli) [38], and presumed intentionality (imagined to be evaluating the viewer vs. someone else) [19, 39]. Similar modulations occur at the P300, which is larger to shifts of gaze when participants believe that computer-generated faces are controlled by a real person [20, 40, 41]. These examples highlight that the same facial information can elicit different neural responses based on context, even at early perceptual stages, and that these ERPs are effective in indexing the neural processing of eye-contact and its modulation by context.

The N170 and P300 have been the focus of much research investigating the neural bases of social cognition in ASD. The most consistent finding is that people with ASD show delayed N170 latency to faces [42], presumably reflecting processing inefficiency potentially associated with reduced exposure to faces across development [43]. The majority of this research has utilized static images of faces rather than dynamic faces, passive viewing rather than interactive naturalistic viewing, and randomized block designs in which the temporal onset of a face is devoid of context (e.g., predictable versus unpredictable behavior). Though some of these design choices reflect recently resolved methodological limitations that precluded dynamic and interactive ERP [44], [36] experiments, the literature currently offers little information about potentially meaningful individual differences in neural dynamics associated with dynamic, interactive faces across predictive contexts.

In this study, we evaluate the hypothesis that neural response to eye-contact in ASD is differentially influenced by the predictability of social context. Toward this end, we co-registered EEG with simultaneous eye tracking (ET) in an innovative experimental paradigm that simulated face-to-face interaction with onscreen faces responsive to a participant’s eye gaze. This enabled us to explicitly manipulate the predictability of receiving eye-contact. Across two experiments, we presented virtual social partners engaging in reciprocal eye-contact in an unpredictable or predictable context. We evaluated two possibilities: (1) In predictable contexts, participants with ASD would show comparable responses, e.g., in modulation of the N170 and P300, to typically developing adults. However, in unpredictable contexts, we hypothesized that individuals with ASD would show enhanced N170s and P300s, reflecting increased sensitivity to unpredictable contexts. This possibility is consistent with manipulations of predictability of gaze perception in ASD. Alternatively, (2) it is possible that across both contexts, people with ASD would show attenuated and delayed response to eye-contact, consistent with prior research, which hypothesizes a primary deficit in social perception (e.g., faces and eyes) in ASD. Finally, because many people with ASD report eye-contact as anxiety-inducing and/or distracting, we conducted correlative analyses to examine whether these neural responses to eye-contact were associated with anxiety or visual sensory sensitivity.

## Methods and materials

### Participants

Participants included 23 (Exp 1) and 25 (Exp 2) adults with ASD and 46 (Exp 1) and 43 (Exp 2) typically developing (TD) participants recruited from the greater New Haven area and screened for psychiatric conditions. Participants with ASD were diagnosed using gold standard research tools, including the Autism Diagnostic Observation Schedule 2nd Edition (ADOS-2) [45] administered by a research-reliable administrator and DSM-5 diagnosis by a licensed clinician. Social communicative symptomology was measured by the ADOS-2 Calibrated Severity Score (CSS), anxious symptomology was measured using the Beck Anxiety Inventory (BAI) [46], and ASD-related visual sensory sensitivities were measured using the visual subscale of the Glasgow Sensory Questionnaire (GSQ) [47], a self-report measure specifically designed for characterizing ASD sensory symptomology. Participant characteristics are shown in Table 1. Exclusionary criteria included current use of benzodiazepine or anticonvulsant medication; a history of seizures or head injuries; substance abuse or dependency; primary psychiatric diagnosis that was not ASD; full scale IQ < 70, as measured by the Wechsler Abbreviated Scale of Intelligence 2nd Edition (WASI-II) [48]; or other factors that would preclude successful recording of eye tracking and EEG. Additionally, TD participants were excluded if they had psychiatric/neurodevelopmental conditions or a first degree relative with an ASD diagnosis. Participants, or their legal guardians, provided written informed consent and received financial compensation for participation. The study was approved by the institutional review board at the Yale University School of Medicine.

**Table 1:** Demographic and clinical characteristics in ASD and TD controls

	DX	*N*	IQ (SD)	Age (SD)	% Female
Exp 1	ASD	23	106 (18.8)	23.08 (5.74)	0.22
	TD	46	112.33 (14.5)	26.53 (6.35)*	0.43
Exp 2	ASD	25	104.6 (18.2)	24.49 (5.88)	0.2
	TD	43	112.07 (15.4)	27.27 (6.60)	0.44

*p < .05

### Experimental tasks

Adults participated in one of two experiments designed to measure brain response to interactive eye-contact. We used high-speed eye tracking to measure where a participant looked on a computer screen. The location of looking was integrated, in real time, with the experimental control software so that the experiment could react to where the participant was looking. In this way, onscreen faces would change in response to being looked at, giving the impression of reciprocity. In both experiments, a single facial feature changed in response to participant fixation, giving the impression of apparent facial movement.

In both experiments, onscreen faces could respond to participant fixation with reciprocal eye-contact. However, the experiments differed in whether participants could accurately predict the receipt of eye-contact. In the first experiment, the receipt of eye-contact was unpredictable in that the face might respond with eye-contact, or mouth movement. In the second experiment, the face would change between direct and averted gaze, allowing the participant to accurately anticipate when they would receive eye-contact.

#### Experiment one: unpredictable eye-contact

**Fig. 1:**
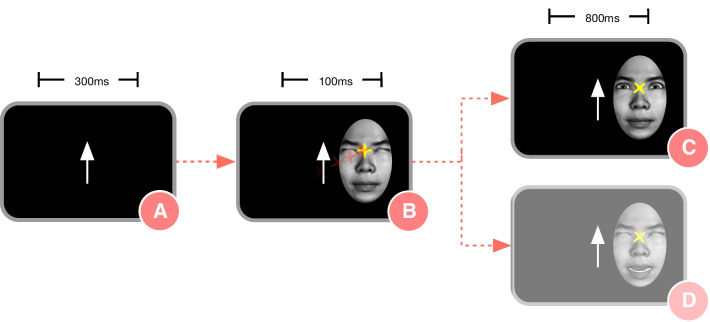
Trial structure for Experiment One. Trial structure for Experiment One: A blank screen was followed by **A** a centrally presented arrow pointing up or down, cueing participants to look to the eyes (up) or mouth (down) of the subsequently appearing face; **B** a peripherally presented face with eyes and mouth closed; contingent on participant fixation to the cued region (eyes or mouth), the face responded by opening its eyes (**C**) or mouth (**D**). The yellow “X” indicates an example participant point of fixation for the illustrated trial

Trials began with a centrally presented onscreen fixation arrow (pointing up or down; Fig. 1A). Contingent upon participant fixation to the arrow for 300 ms, a peripherally presented face with closed eyes and mouth appeared on screen. As per verbal and written instructions provided to the participant prior to the task, fixation arrows cued the participant to look either to the mouth (arrow pointing down) or the eyes (arrow pointing up; Fig. 1B) of the subsequently appearing face. Contingent upon participant fixation for 100 ms to the face, the face responded by either opening its eyes (Fig. 1C) or mouth (Fig. 1D), and then remained on screen for 800 ms. In this way, four types of face-to-face interactions were displayed, with 57 trials per type: (1) the participant looks to the eyes and the eyes open (reciprocal eye-contact, eye:eye), (2) the participant looks to the mouth and the mouth opens (mouth:mouth), (3) the participant looks to the mouth and the eyes open (mouth:eye), or (4) the participant looks to the eyes and the mouth opens (eye:mouth). Only reciprocal (eye:eye, mouth:mouth) trials were considered for current analyses. Experiment One stimuli consisted of grayscale digital images of neutral faces with their eyes and mouths closed and open; images were generated by the FaceGen software package and rendered using the Softimage software package. Faces were masked in an oval frame to remove non-face features [49]. Importantly, because both the eyes and mouth of the face could open on any trial, participants could not predict receipt of eye-contact, enabling us to assess neural response to reciprocal eye-contact under *unpredictable* conditions.

#### Experiment two: predictable eye-contact

**Fig. 2:**
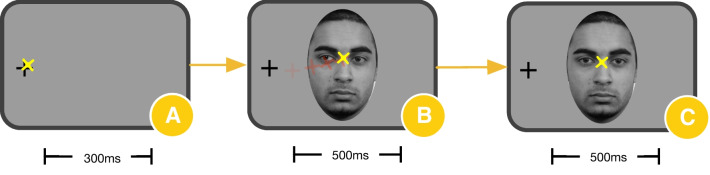
Trial Structure for Experiment Two. Trial structure for Experiment Two: Crosshairs were initially presented to the left or right side of the screen (**A**); contingent on participant fixation to the crosshair for 300 ms, a centrally presented face appeared displaying direct or averted gaze (**B**); contingent on participant fixation to the eyes of the face for 500 ms, the face changed gaze from direct to averted or averted to direct as depicted in **C**. Stimulus change was confined to the eyes of the face. The yellow “X” indicates example participant point of fixation

Trials began with a peripherally presented fixation cross on the left or right side of the screen (Fig. 2A). Contingent upon participant looking to the fixation cross for 300 ms, a centrally presented face appeared displaying either direct or averted gaze (Fig. 2B). Contingent on participant fixation to the eyes of the face for 500 ms, the face changed its gaze from averted to direct (eye-contact) or from direct gaze to averted (averted gaze) and remained on screen for 600 ms (Fig. 2C). There was a total of 45 trials in each condition. Stimuli consisted of grayscale images of static adult faces displaying both direct and averted gaze from the Radboud Faces Database [50]. Faces were masked in an oval frame to remove non-face features [49]. In contrast to Experiment One, because all movement was constrained to the eyes of the onscreen face, participants could always predict receipt (or disengagement) of eye-contact, enabling us to assess neural response to reciprocal eye-contact under *predictable* conditions.

### Data acquisition and processing

EEG data were recorded at 1000 Hz from an EGI NetAmps 300 amplifier running Net Station 4.5 acquisition software. EEG was collected from an EGI 128-channel Hydrocel Geodesic Sensor Net. Impedances were kept beneath 40 kilo-ohms. Eye tracking data were collected from the right eye with an SR-research Eyelink 1000 eye tracker in remote mode at 500 Hz. Calibration was collected at the start of the experiment with a 13-point calibration, and all participants were calibrated within 2 degrees of error. Experimental presentation was controlled by a separate computer using PsychToolbox and Eyelink Toolbox experimental control software to ensure accurate timing and synchronization between EEG and eye tracking systems.

To calculate ERPs, raw EEG data was processed to a robust average reference, and bad channels were interpolated using the PREP Pipeline [51] and filtered from 0.01 to 00 Hz. EEG was segmented to the onset of facial change from − 100 to 500 ms post-facial change. Artifactual segments were excluded if they contained channels with a range greater than 100 mV. All participants had at least 25 artifact-free trials per experimental condition. The N170 was extracted from right occipital electrodes conforming to T6 in the 10–20 system using electrodes (83, 84, 85, 89, 90, 91, 95, 96) on the Hydrocel 128-channel net. The P300 was extracted from central electrodes (80, 55, 31, 61, 62, 79, 72, 53, 78). N170 peak latency and amplitude were selected in the range from 130 to 250 ms. Our choice to use peak, rather than mean, measures of N170 activity were driven by precedent in the literature for measuring the peak of the N170, and evidence that the latency of the N170 peak reflects unique information regarding the processing of social information in ASD [33, 36, 42, 52–54]. The P300 was measured as the mean amplitude between 300 and 410 ms. We quantified mean, rather than peak, amplitude from the P300, as prior literature examining dynamic faces has revealed highly variable waveform morphologies that do not exhibit well defined peaks across all individuals. [36, 55, 56] ERP processing was conducted in the EEGlab [57] and ERPlab [58] toolboxes. All analyses were run using the R statistical programming language [59] using the AFEX package. [60]

## Results

**Fig. 3:**
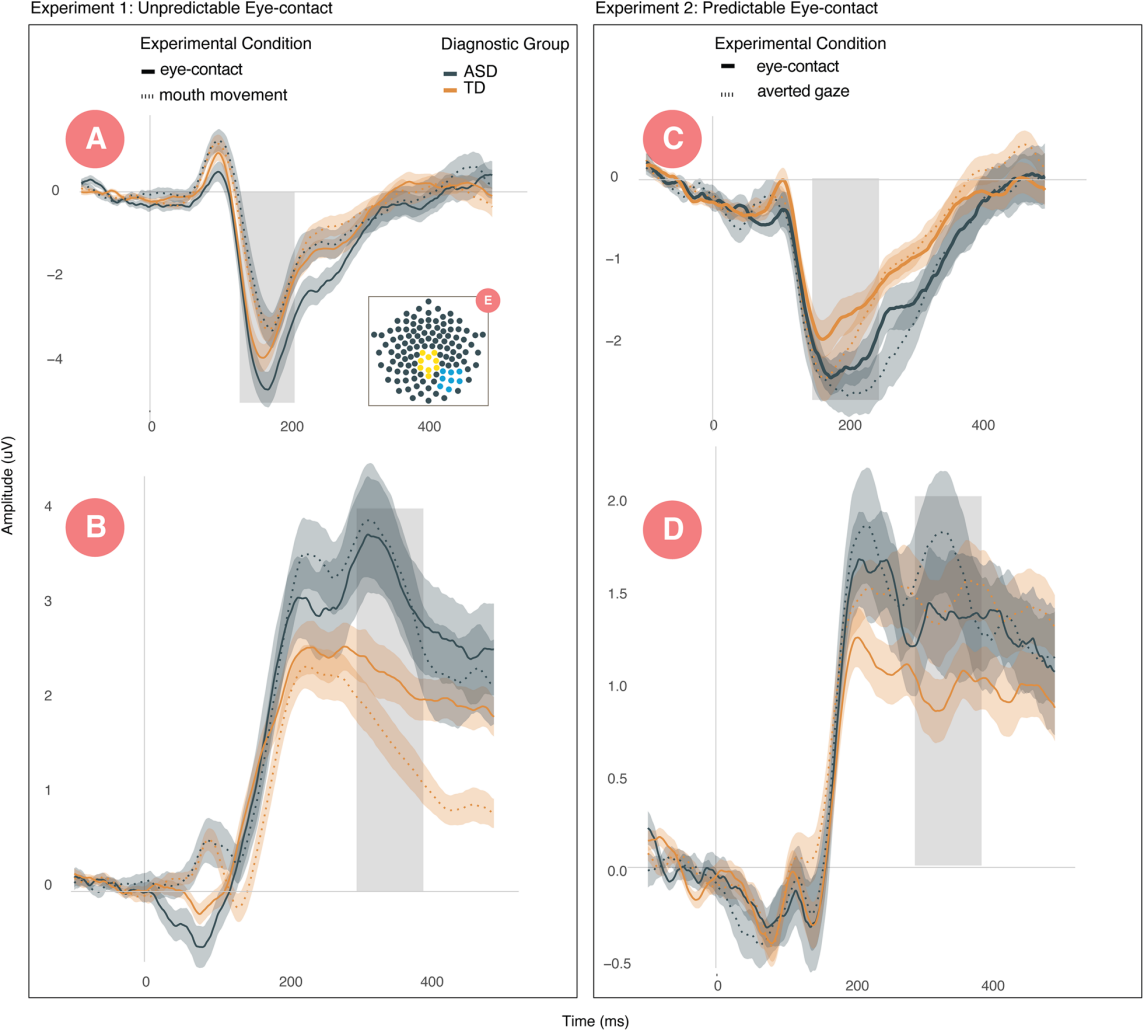
Waveforms for Experiments One and Two. The left panel presents **A** N170 and **B** P300 for unpredictable eye-contact. The right panel presents **C** N170 and **D** P300 for predictable eye-contact. The inset electrode layout **E** depicts the electrodes averaged to extract the N170, in blue, and the P300, in yellow. Gray regions demarcate the temporal range for component extraction. Line colors indicate diagnostic group (ASD/TD), and line style (solid/dashed) indicates experimental contrasts. Confidence intervals around waveforms reflect standard errors

To assess neural response to eye-contact, separate univariate repeated measures ANOVAs with experimental condition as a within-subjects factor and diagnostic group as a between-subjects factor were run for each experiment and ERP component (N170, P300). Results of repeated measures ANOVAs are shown in Table 2. To estimate the relationships between differential processing of eye-contact and clinical characteristics, we calculated difference scores between experimental conditions at both the N170 (amplitude and latency) and P300 and correlated them with ADOS-2-CSS, BAI, and GSQ scores. Descriptive statistics for all scores are presented in Additional file 1. We also estimated correlations with overall N170 latency to faces as this marker has been informative in prior research. Grand averaged ERP waveforms are shown in Fig. 3.

**Table 2:** Results from repeated measures ANOVAs for experiments one and two

Term	DF	*F*	*η*^2^	*p*	FDR adjusted *p*
EXP 1 unpredictable eye-contact					
*N170 amplitude*					
DX group	67	0.81	0.01	0.371	0.555
condition	67	34.69	0.34	**0.001**	**0.001**
DX x condition	67	4.70	0.07	**0.034**	0.093
*N170 latency*					
DX group	67	0.59	0.01	0.444	0.615
condition	67	11.73	0.15	**0.001**	**0.021**
DX x condition	67	1.33	0.02	0.254	0.542
*P300 amplitude*					
DX group	67	9.09	0.12	**0.004**	**0.034**
condition	67	9.98	0.13	**0.002**	**0.028**
DX x condition	67	6.70	0.09	**0.012**	0.057

EXP 2 predictable eye-contact					
*N170 amplitude*					
DX group	66	3.75	0.05	0.057	0.139
condition	66	9.87	0.13	**0.003**	**0.032**
DX x condition	66	0.99	0.01	0.323	0.554
*N170 latency*					
DX group	66	8.13	0.11	**0.006**	**0.043**
condition	66	1.14	0.02	0.290	0.623
DX x condition	66	5.34	0.07	**0.024**	0.086
*P300 amplitude*					
DX group	66	0.54	0.01	0.464	0.623
condition	66	5.03	0.07	**0.028**	0.086
DX x condition	66	0.62	0.01	0.434	0.651

Bold values indicate *p* < .05

### Experiment one: unpredictable eye-contact

In Experiment One, there were main effects of experimental condition for all components such that eye-contact, relative to mouth movement, yielded more negative [*F*(1,67) = 34.7, *p* < 0.001)] and earlier [*F*(1,67) = 7.6, *p* = 0.007)] N170s and more positive P300s [*F*(1,67) = 9.98, *p* = 0.002)]. Additionally, there was a main effect of diagnosis at the P300, such that individuals with ASD demonstrated larger P300s, across experimental conditions, compared to controls [*F*(1,67) = 9.1, *p* = 0.003]. Finally, there were significant interaction effects, such that, relative to controls, individuals with ASD were more sensitive to unpredictable eye-contact at the N170 but less sensitive at the P300. These interaction effects revealed that individuals with ASD showed a greater difference between eye-contact and mouth movement at the N170 [*F*(1,67) = 4.6, *p* = 0.033] but a smaller difference between eye-contact and mouth movement at the P300 [*F*(1,67) = 6.7, *p* = 0.01].

Increased N170 amplitude to eye-contact compared to mouth movement correlated with anxiety as measured by the BAI (*r* =  − 0.31, *p* = 0.027) and visual sensitivity as measured by the GSQ (*r* =  − 0.36, *p* = 0.008), such that individuals with greater response to eye-contact reported increased anxiety and visual symptomology. Increased P300 amplitude to eye-contact relative to mouth movement was associated with lower autism symptoms on the ADOS-2-CSS (*r* =  − 0.34, *p* = 0.009) and lower levels of anxiety (*r* =  − 0.30, *p* = 0.032).

### Experiment two: predictable eye-contact

In Experiment Two, a main effect of condition indicated that predictable reciprocal eye-contact evoked less negative [*F*(1,66) = 9.8, *p* = 0.002] N170s and less positive P300s [*F*(1,66) = 5.0, *p* = 0.028)] than averted gaze. Additionally, there was a main effect of diagnostic group on N170 latency such that individuals with ASD, relative to TD, demonstrated longer N170 latency in both experimental conditions [*F*(1,66) = 8.1, *p* = 0.005]. An interaction effect indicated that individuals with ASD exhibited slower N170s to averted gaze, relative to direct gaze, compared to controls [*F*(1,66) = 5.3, *p* = 0.024)]. There were no main effects or interactions with diagnosis at the P300.

In Experiment Two, decreased N170 latency to eye-contact relative to averted gaze and N170 latency were associated with increased autism symptoms as measured by the ADOS-2-CSS (*r* = 0.31, *p* = 0.01; *r* = 0.3, *p* = 0.02, respectively).

## Discussion

In this study, we assessed whether predictability of interactive eye-contact influenced neural response in ASD. When the receipt of eye-contact was unpredictable, people with ASD, compared to controls, showed stronger initial neural responses to eye-contact relative to mouth movement with increased N170s and indiscriminate later response as identified by increased amplitude P300s to any facial movement but no specificity to the type of facial movement, as indicated by the absence of a group by condition interaction. Conversely, when the receipt of eye-contact was predictable, individuals with ASD showed expected patterns of delayed N170 to eye movement, with delays more pronounced for averted relative to direct gaze and no difference at the P300. These findings demonstrate (1) that people with ASD integrate contextual information to bias neural response to faces at the earliest neural levels of gaze processing and (2) that this bias is different than that of healthy controls.

In addition to group differences, and consistent with reports of eye-contact being distracting and/or anxiety-inducing brain activity was related to socio-emotional and sensory function across diagnostic groups in both experiments. These relationships also varied between unpredictable and predictable contexts. In unpredictable contexts, the N170 was associated with anxiety and sensory sensitivity. Specifically, anxiety was associated with increased response to eye-contact at the N170 and overall increased P300. This pattern of may suggest an overall increased state of arousal in unpredictable situations in ASD that leads to increased sensory sensitivity, e.g., differentiation at the N170 but indiscriminately larger response at the P300. This interpretation is consistent with noradrenergically driven interpretations of anxiety and arousal that are associated with both ASD diagnostic status, sensory function, and the P300 [61–63]. Conversely, in predictable contexts, the N170 was associated with social performance as measured by the ADOS. This pattern of results suggests that the N170, which represents the earliest stages of face and gaze processing, may be modulated by input from upstream frontal neural systems that flexibly potentiate or attenuate early social perception based on available information. If so, characterization of N170 activity under different task demands may provide a unique and valuable source of information for biomarker development in ASD. Interestingly, the P300 in unpredictable contexts, at which individuals with ASD did not show differentiation between conditions, was also associated with impairments in social performance as measured by the ADOS. This distinction demonstrates that while individuals with ASD can be hypersensitive to socially relevant changes in gaze at some components, i.e., the N170, this sensitivity does not confer reduced symptomology. In fact, the pattern of relationships in unpredictable situations suggests that activity driven by eye-contact at early perceptual levels, e.g., the N170 may serve to impede later cognitive processes, e.g., reducing neural discrimination at the P300.

Our results also inform the heterogeneity of findings of prior ERP research in face processing in ASD. Delays at the N170 to faces are one of the most replicated findings in ASD, but they are not universally identified [42]. Here we find that these delays are only present under conditions in which individuals with ASD view eye-contact in predictable social contexts. Unpredictable social contexts, in contrast, showed potentiated instead of attenuated processing. In placing our findings in the context of prior research, it is important to highlight several meaningful advances between our approach and those employed in most prior studies. First, using eye tracking, we ensured that all participants were looking to the eyes of the face. Fixation to eyes elicits larger and earlier N170s, and although this effect is diminished in ASD [52], our methods effectively eliminated this source of potential variation between groups. Secondly, we estimated ERPs to facial movement, rather than to the appearance of a face. Because different cortical regions respond selectively to static vs. dynamic facial images [64, 65], we may have selectively modulated function in specific cortical contributors to the N170. Finally, we presented faces in a dynamic interaction initiated by the participant with their gaze. In this way, for both predictable and unpredictable contexts, participants were not passive observers. Active attention vs. passive viewing has a robust effect on early visual ERPs [34, 66]. In this way, social interactions may yield a qualitatively different neural signal to eye-contact than is generated from passive observation. Differences from prior literature notwithstanding our findings offer a possible contributory explanation for heterogeneous ERP findings in face processing research in ASD that have not explicitly addressed the modulatory role of context effects on gaze perception. In manipulating the predictability of the social context, we show that gaze processing is neither globally attenuated nor potentiated but is differentially sensitive to context in ASD.

## Limitations

These results should be interpreted in the context of the following limitations. First, our manipulation of context and predictability was limited to completely predictable and completely unpredictable facial movements. These artificial extremes, while useful for our experimental investigation, fail to represent the probabilistic conditions that exist in real life. Secondly, our sample included adults with IQ > 70. Many individuals with ASD have IQ < 70, and further research is needed to understand how the results here are generalizable across the spectrum of cognitive function and age. Additionally, our manipulation of predictability across our experiments is conflated with stimulus contrasts for eye-contact. In Experiment One, we compare eyes to mouths. In Experiment Two, we compare eyes to eyes. These experiments differ in the magnitude of the visual change of eye-contact between experiments, i.e., the differences between gaze shifts entail less overall motion than eyes opening; we note that this visual difference was quite small, subtending < 2.5 degrees of visual angle. While it is possible that our experimental differences were, in part, driven by these low-level visual changes, these stimuli differences would not account for the observed interaction effects involving diagnostic groups. Furthermore, despite this potential confound, prior research supports our interpretation that context, rather than content (e.g., direct vs. averted gaze), has a potent influence on neural response [18, 38–40, 66] to facial movement as similar comparisons (e.g., eye vs. mouth movement) have failed to identify difference in non-interactive contexts [56]. Nevertheless, the potential contribution of low-level visual differences in the effects studied here should be explored more specifically in future research. It is also important to point out that we report here uncorrected p values for our statistical tests. While all analyses were grounded in a-priori hypotheses, our quantification of continuous symptom relationships with brain activity was hypothesis-generating and should be re-evaluated in future hypothesis-driven work. In particular, not all tests survived false-discovery correction and adjusted p values are presented in Tables 2 and 3 alongside measures of effect size. As these tables show, while many results remain significant, many others, particularly relationships between clinical characteristics and brain response, do not remain significant. Moreover, a particularly important limitation of this study was the inclusion of only four autistic women. Autistic women are historically underrepresented in research and only recently have there been concerted research efforts to correct this bias [67–70]. This underrepresentation is relevant especially as it pertains to the relationship of clinical characteristics, such as anxiety, which are known to exhibit differential prevalence and symptom profile in the non-ASD population. While the small number of autistic women in our sample precludes meaningful analyses of this subgroup, include a supplemental analysis with sex assigned at birth as a factor. As the sex differences between groups largely tracked with diagnostic status, these analyses yield similar findings although in some cases the effect of diagnostic group is attenuated. As these results in the supplement show, it is critical that research proactively address the issues of representation from the outset via targeted recruitment and oversampling of underrepresented groups, rather than attempt to statistically correct or account for these differences if groups are imbalanced [71]. Finally, it is important to highlight that, in this experiment, participants were cued to look to the eyes of faces and many people with ASD may not, in fact, attend to the eyes of faces in naturalistic or un-cued situations [72]. However, many people with ASD have had significant exposure to interventions wherein looking to the eyes is preceded by an explicit behavioral cue, e.g., applied behavior analysis (ABA) or pivotal response treatment (PRT). In this way, our unpredictable context may represent a specific violation of prior experience for some participants with ASD who have had prior experience with cued attention to faces. A replication of our experimental design with precise characterization of participant intervention history is necessary to explore this hypothesis further.

**Table 3:** Correlations between neural response and clinical characterization

	ampdif	latdif	lat	P300	CSS	BAI	GSQ
*Exp 1*							
ampdif		− 0.02	0.05	0.11	− 0.25	− 0.31	− 0.36
latdif	*0.84*		0.04	0.00	0.05	− 0.06	− 0.04
lat	*0.66*	*0.74*		− 0.09	0.12	0.03	− 0.13
P300	*0.39*	*0.97*	*0.45*		− 0.34	− 0.30	− 0.24
CSS	*0.06*	*0.72*	*0.37*	*0.01*		0.56	0.32
BAI	*0.03*	*0.65*	*0.82*	*0.03*	< *.001*		0.55
GSQ	*0.01*	*0.77*	*0.35*	*0.08*	*0.02*	< *.001*	
*Exp 2*							
ampdif		− 0.25	− 0.08	0.64	0.00	− 0.01	− 0.06
latdif	*0.84*		− 0.25	0.11	− 0.31	− 0.06	− 0.14
lat	*0.05*	*0.04*		0.11	0.29	0.12	0.01
P300	*0.59*	*0.36*	*0.38*		0.14	0.08	0.00
CSS	*0.98*	*0.02*	*0.03*	*0.29*		0.50	0.31
BAI	*0.92*	*0.65*	*0.37*	*0.58*	< *.001*		0.67
GSQ	*0.66*	*0.32*	*0.94*	*0.99*	*0.03*	< *.001*	
*Exp 1 FDR Adjusted*							
ampdif		− 0.02	0.05	0.11	− 0.25	− 0.31	− 0.36
latdif	0.84		0.04	0.00	0.05	− 0.06	− 0.04
lat	0.66	0.74		− 0.09	0.12	0.03	− 0.13
P300	0.39	0.97	0.45		− 0.34	− 0.30	− 0.24
CSS	***0.14***	***0.84***	***0.56***	***0.05***		0.56	0.32
BAI	***0.09***	***0.79***	***0.90***	***0.09***	< *.001*		0.55
GSQ	***0.05***	***0.87***	***0.56***	***0.19***	*0.02*	< *.001*	
*Exp 2 FDR Adjusted*							
ampdif		− 0.25	− 0.08	0.64	0.00	− 0.01	− 0.06
latdif	*0.84*		− 0.25	0.11	− 0.31	− 0.06	− 0.14
lat	*0.05*	*0.04*		0.11	0.29	0.12	0.01
P300	*0.59*	*0.36*	*0.38*		0.14	0.08	0.00
CSS	***0.99***	***0.07***	***0.08***	***0.54***		0.50	0.31
BAI	***0.98***	***0.78***	***0.55***	***0.75***	< *.001*		0.67
GSQ	***0.78***	***0.55***	***0.88***	***0.99***	*0.03*	< *.001*	

Pearson’s *r* values are shown on the upper diagonal, and *p *values are shown on the lower diagonal. Italic cells on the lower diagonal table indicate FDR adjusted *p *values. Bold cells indicate relationships between characterization measures and brain activity

## Conclusion

In summary, we found that neural response to interactive eye-contact in ASD ranges from attenuated to hypersensitive depending on the social context. The social world can be unpredictable, and our results show that for some people with ASD, eye-contact can be differentially modulated by this unpredictability, fitting with multiple theories [23, 25, 26, 73]. Extant research has demonstrated that people with ASD show differences in how they integrate context into *sensory* perception and here we show that (1) these effects act on *social* perception; (2) during simulated social interactions; and (3) on a time course commensurate with actual social interactions. These results help resolve the discrepancy between why some people might struggle with gaze processing in everyday life but show no challenges in interventions or research studies. It may be that the perception of eye-contact in ASD, and vulnerabilities therein, emerges in the moments *before* eye-contact is made. Eye-contact and social gaze are fast, fleeting, and unpredictable. Thus, if someone is not prepared to use eye-contact as a social tool in the rapid back and forth of a social interaction, then it changes from a useful tool to an ever-present impediment. In this way, we enrich our understanding of eye-contact vulnerabilities in ASD and bridge ostensibly non-social theories of cognition in ASD which have identified differences in prediction, habituation, and learning across a variety of domains, and social theories which are diagnostically more face-valid and the target of treatments, but vastly more difficult to circumscribe within an experimental context. Eye-contact in a social interaction, unlike a static face on a computer screen, occurs on a rich and dynamic backdrop of prior experience and expectations. Here, we show that, even under simple manipulations of those expectations, perception of eye-contact changes dramatically in people with ASD. This finding highlights the need for further explorations of the underlying biology guiding perceptual processing and prediction in ASD, as well as a more rigorous examination and quantification of social behavior itself. Understanding how people use contexts to modulate social perception is only helpful inasmuch as we can know when they can, or cannot, employ that modulation.

## Supplementary Information

**Additional file 1**. Supplemental analyses.

## Data Availability

Datasets generated and/or analyzed during the current study are available from NIMH NDA (#2312):
