# Supplementary material for: Predictability modulates neural response to eye contact in ASD
Source: Mol Autism. 2022 Oct 29;13:42. doi: 10.1186/s13229-022-00519-0 (PMC9618208; doi:10.1186/s13229-022-00519-0)
Supplement: Supplementary file 1 — Additional file 1. Supplemental analyses. [file 13229_2022_519_MOESM1_ESM.pdf]

Supplement 1: Exploration of Sex, Proportion of artifact free trials, and IQ on dependent measures

1. Analytic models including Sex as a factor.

Table 1*: Experiment 1: N170 Amplitude including Sex*

	num Df	den Df	MSE	F	pes	Pr(>F)
DX	1.000	65.000	7.228	0.001	0.000	*0.971*
Sex	1.000	65.000	7.228	1.933	0.029	*0.169*
DX:Sex	1.000	65.000	7.228	2.257	0.034	*0.138*
condition	1.000	65.000	1.020	19.519	0.231	*0.000*
DX:condition	1.000	65.000	1.020	1.232	0.019	*0.271*
Sex:condition	1.000	65.000	1.020	1.307	0.020	*0.257*
DX:Sex:condition	1.000	65.000	1.020	2.389	0.035	*0.127*

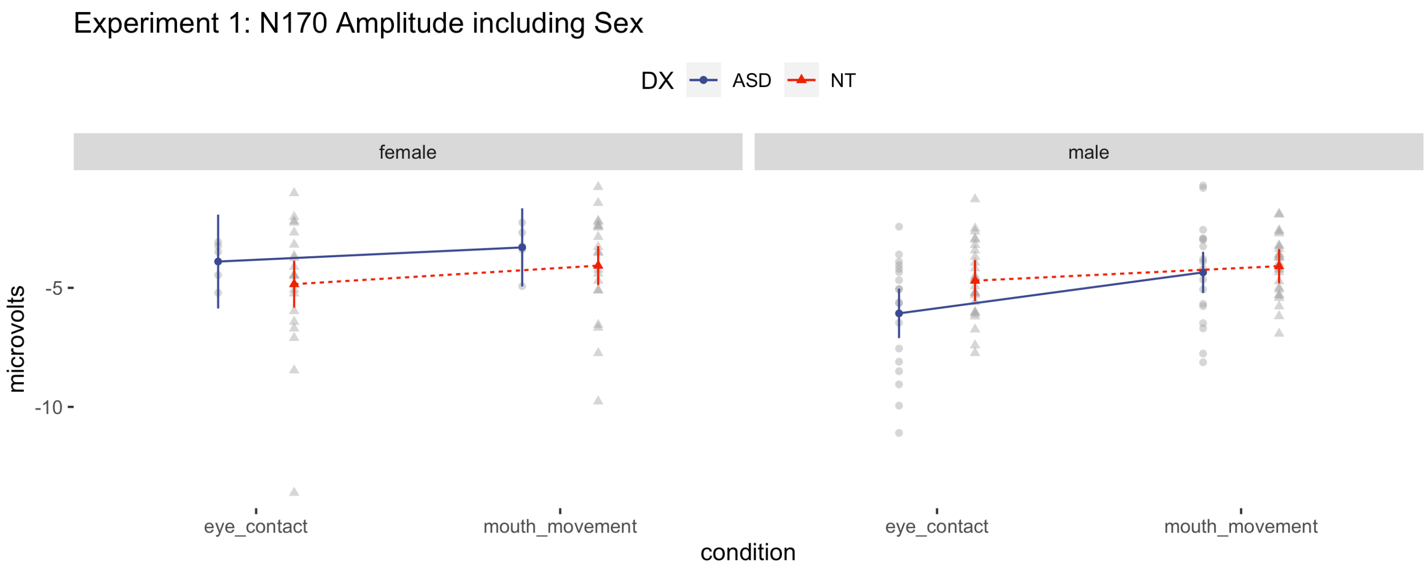

***Table 2**: Experiment 1: N170 Latency including Sex*

	num Df	den Df	MSE	F	pes	Pr(>F)
DX	1.000	65.000	542.801	0.407	0.006	*0.526*
Sex	1.000	65.000	542.801	2.321	0.034	*0.132*
DX:Sex	1.000	65.000	542.801	0.652	0.010	*0.422*
condition	1.000	65.000	123.084	6.902	0.096	*0.011*
DX:condition	1.000	65.000	123.084	2.094	0.031	*0.153*
Sex:condition	1.000	65.000	123.084	0.079	0.001	*0.779*
DX:Sex:condition	1.000	65.000	123.084	1.246	0.019	*0.268*

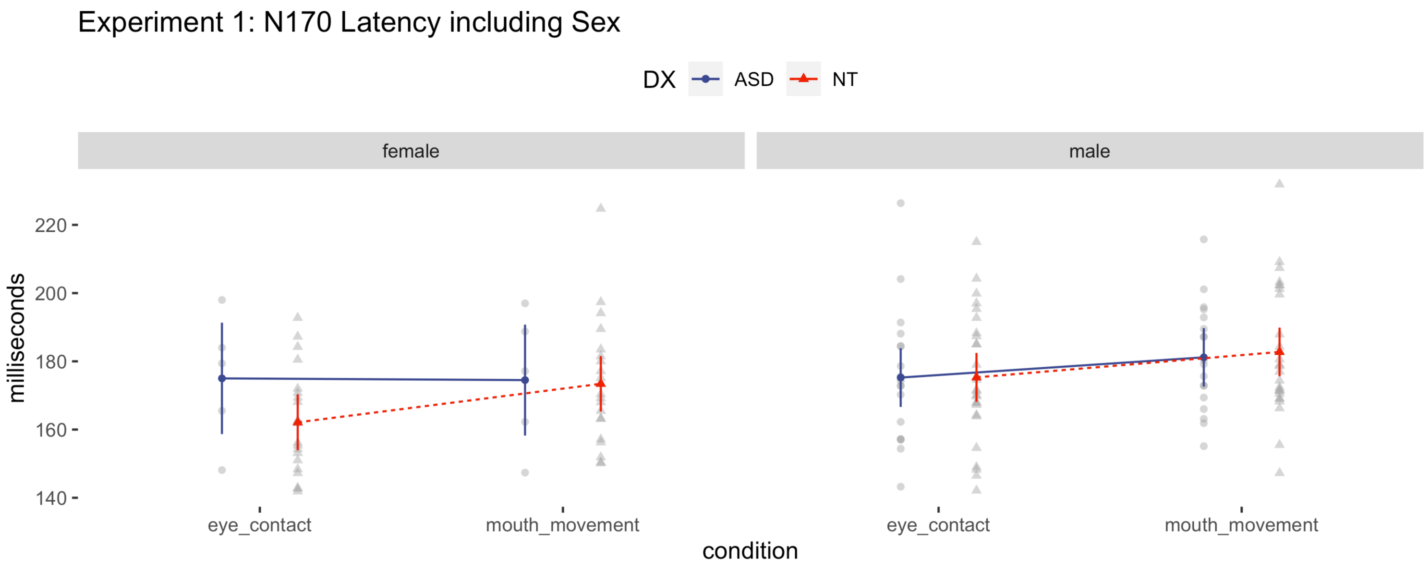

Table 3: *Experiment 1: P300 Amplitude including Sex*

	num Df	den Df	MSE	F	pes	Pr(>F)
DX	1.000	65.000	6.591	6.875	0.096	*0.011*
Sex	1.000	65.000	6.591	0.015	0.000	*0.903*
DX:Sex	1.000	65.000	6.591	0.001	0.000	*0.981*
condition	1.000	65.000	0.622	7.152	0.099	*0.009*
DX:condition	1.000	65.000	0.622	5.488	0.078	*0.022*
Sex:condition	1.000	65.000	0.622	0.020	0.000	*0.887*
DX:Sex:condition	1.000	65.000	0.622	0.179	0.003	*0.674*

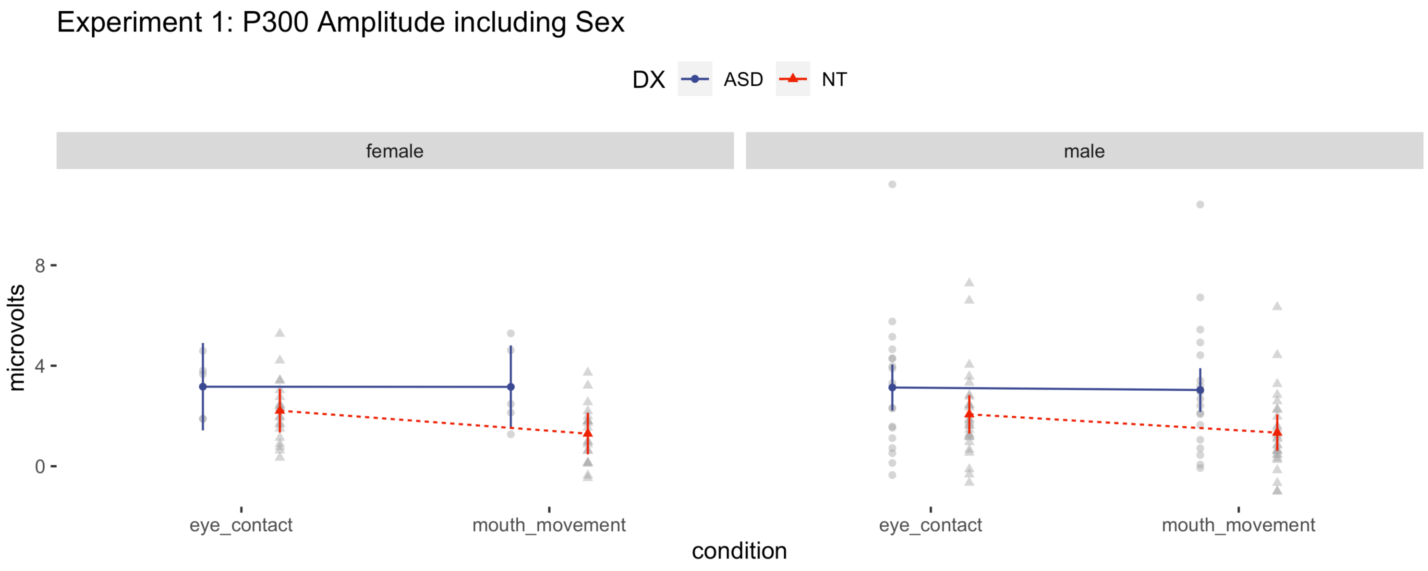

Table 4: *Experiment 2: N170 Amplitude including Sex*

	num Df	den Df	MSE	F	pes	Pr(>F)
DX	1.000	64.000	4.380	3.565	0.053	*0.064*
Sex	1.000	64.000	4.380	0.041	0.001	*0.840*
DX:Sex	1.000	64.000	4.380	0.648	0.010	*0.424*
condition	1.000	64.000	0.446	11.902	0.157	*0.001*
DX:condition	1.000	64.000	0.446	0.002	0.000	*0.969*
Sex:condition	1.000	64.000	0.446	0.924	0.014	*0.340*
DX:Sex:condition	1.000	64.000	0.446	2.697	0.040	*0.105*

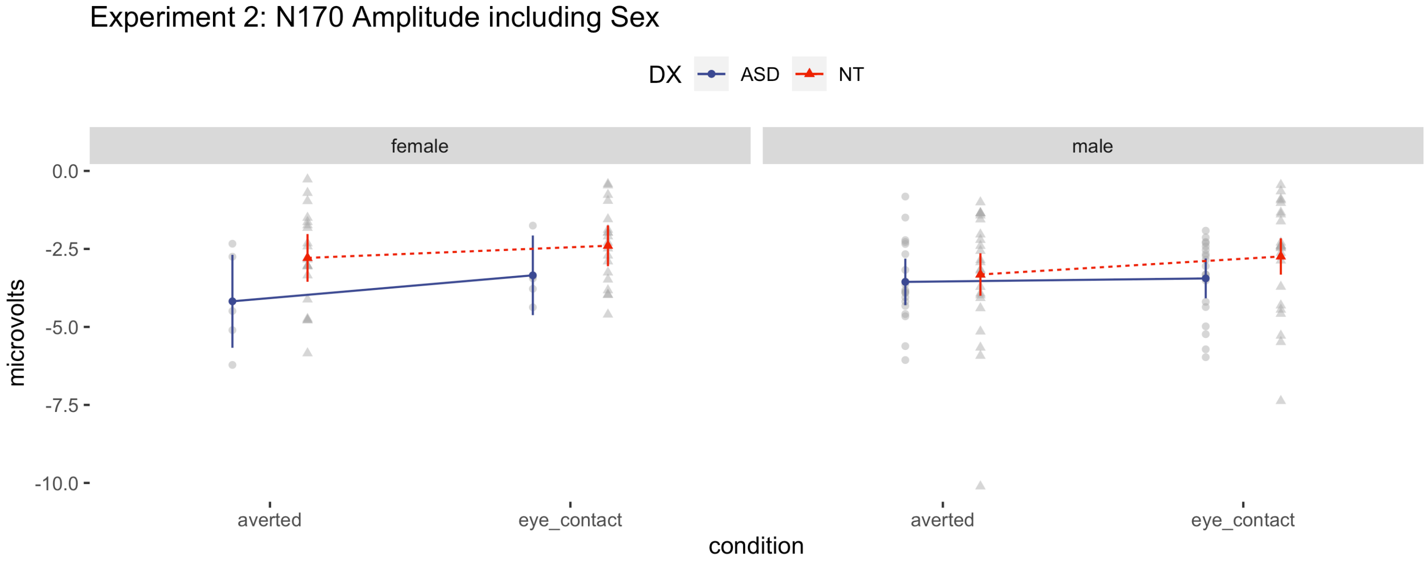

Table 5: *Experiment 2: N170 Latency including Sex*

	num Df	den Df	MSE	F	pes	Pr(>F)
DX	1.000	64.000	677.751	7.970	0.111	*0.006*
Sex	1.000	64.000	677.751	0.852	0.013	*0.359*
DX:Sex	1.000	64.000	677.751	2.264	0.034	*0.137*
condition	1.000	64.000	192.949	0.012	0.000	*0.914*
DX:condition	1.000	64.000	192.949	1.730	0.026	*0.193*
Sex:condition	1.000	64.000	192.949	3.283	0.049	*0.075*
DX:Sex:condition	1.000	64.000	192.949	0.473	0.007	*0.494*

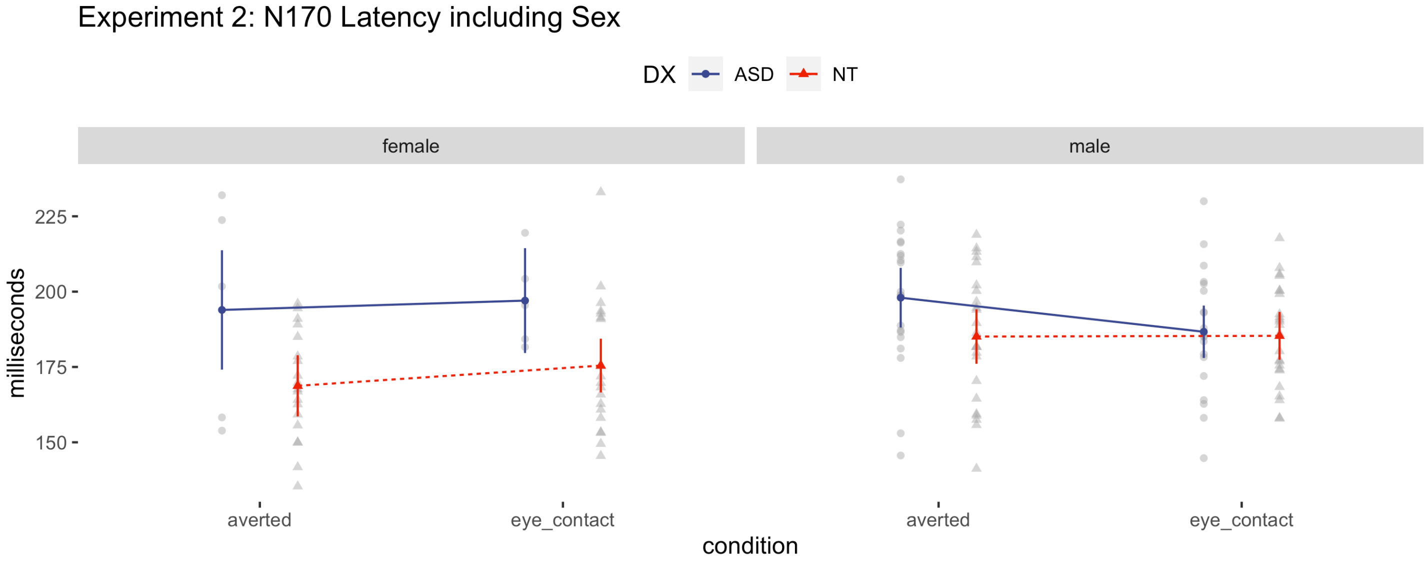

Table 6: *Experiment 2: P300 Amplitude including Sex*

	num Df	den Df	MSE	F	pes	Pr(>F)
DX	1.000	64.000	2.592	0.267	0.004	*0.607*
Sex	1.000	64.000	2.592	0.025	0.000	*0.874*
DX:Sex	1.000	64.000	2.592	0.162	0.003	*0.689*
condition	1.000	64.000	0.832	4.010	0.059	*0.049*
DX:condition	1.000	64.000	0.832	0.215	0.003	*0.644*
Sex:condition	1.000	64.000	0.832	0.045	0.001	*0.832*
DX:Sex:condition	1.000	64.000	0.832	0.510	0.008	*0.478*

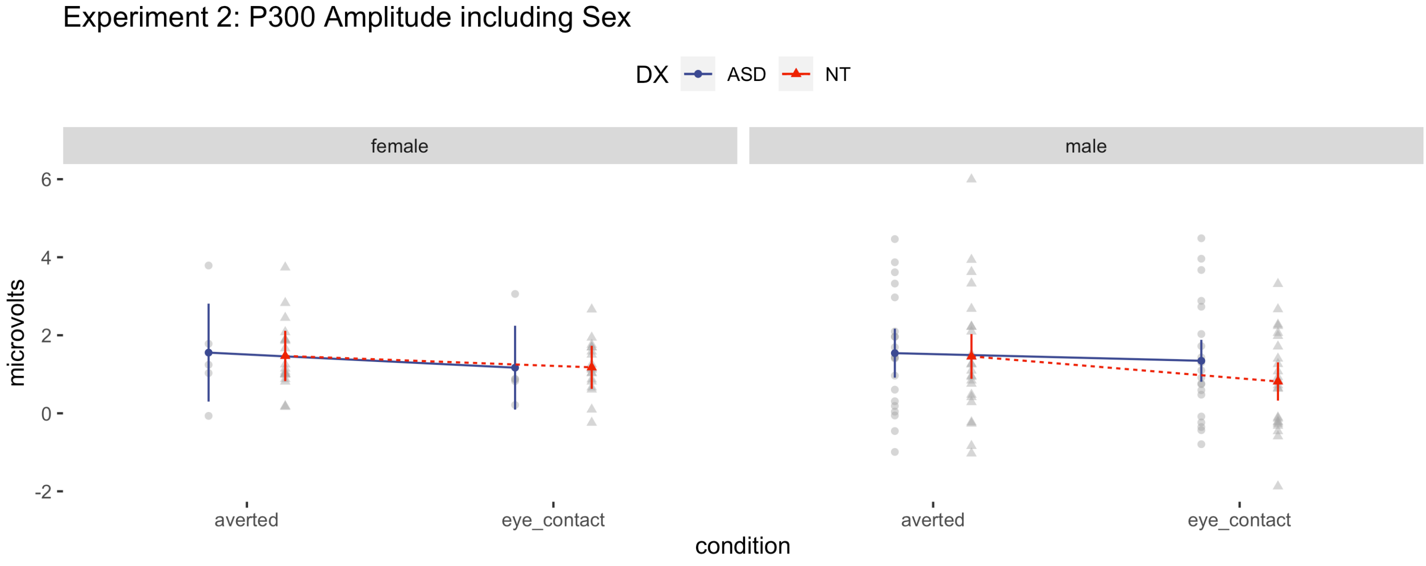

2. Analytic models including # of good trials as a covariate

***Table** 7: Experiment 2: P300 Amplitude with trial count*

	num Df	den Df	MSE	F	pes	Pr(>F)
DX	1.000	64.000	2.349	0.236	0.004	*0.629*
trialCount	1.000	64.000	2.349	5.175	0.075	*0.026*
DX:trialCount	1.000	64.000	2.349	1.479	0.023	*0.228*
condition	1.000	64.000	0.800	0.742	0.011	*0.392*
DX:condition	1.000	64.000	0.800	0.815	0.013	*0.370*
trialCount:condition	1.000	64.000	0.800	0.216	0.003	*0.644*
DX:trialCount:condition	1.000	64.000	0.800	0.331	0.005	*0.567*

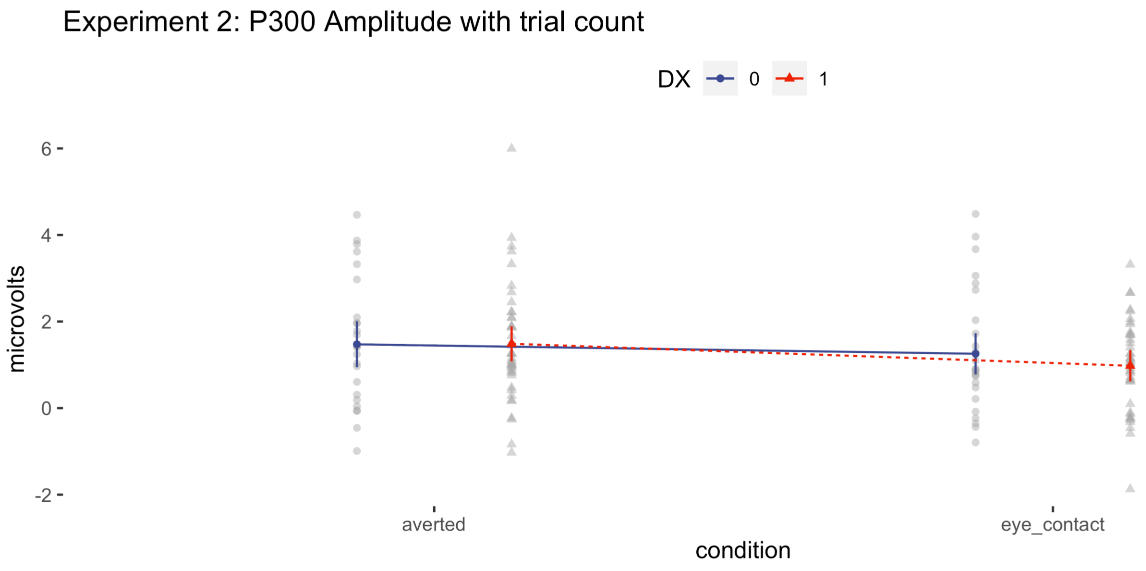

***Table 8**: Experiment 1: P300 Amplitude with trial count*

	num Df	den Df	MSE	F	pes	Pr(>F)
DX	1.000	65.000	6.591	8.619	0.117	*0.005*
trialCount	1.000	65.000	6.591	0.001	0.000	*0.975*
DX:trialCount	1.000	65.000	6.591	0.006	0.000	*0.938*
condition	1.000	65.000	0.591	0.001	0.000	*0.981*
DX:condition	1.000	65.000	0.591	7.982	0.109	*0.006*
trialCount:condition	1.000	65.000	0.591	3.475	0.051	*0.067*
DX:trialCount:condition	1.000	65.000	0.591	3.361	0.049	*0.071*

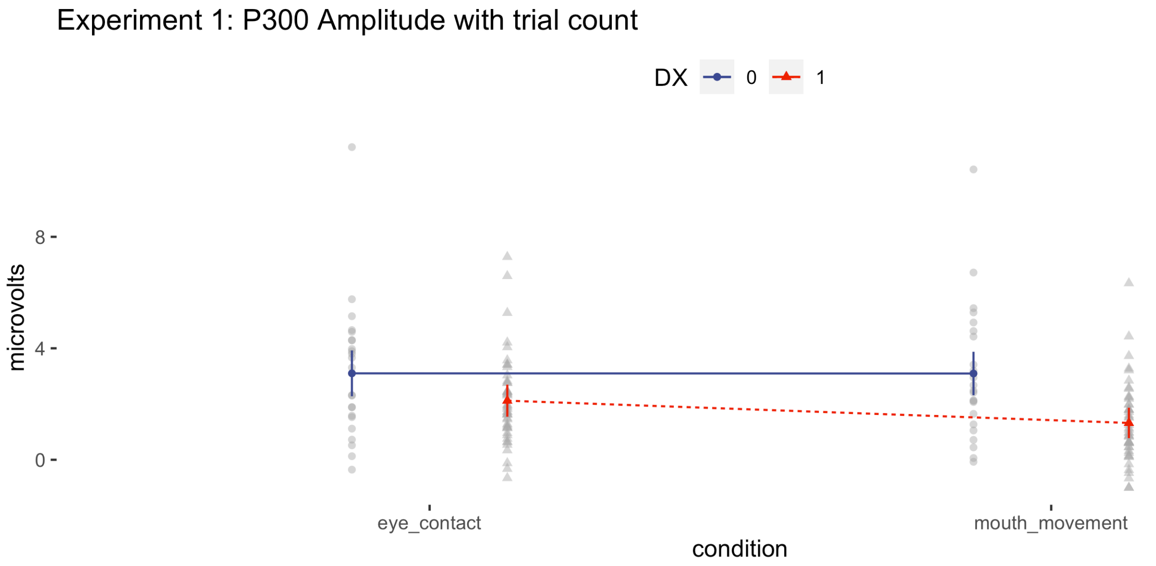

***Table 9**: Experiment 2: N170 Latency with Trial Count*

	num Df	den Df	MSE	F	pes	Pr(>F)
DX	1.000	64.000	734.559	7.807	0.109	*0.007*
trialCount	1.000	64.000	734.559	0.014	0.000	*0.905*
DX:trialCount	1.000	64.000	734.559	0.076	0.001	*0.783*
condition	1.000	64.000	200.735	4.145	0.061	*0.046*
DX:condition	1.000	64.000	200.735	4.876	0.071	*0.031*
trialCount:condition	1.000	64.000	200.735	0.164	0.003	*0.687*
DX:trialCount:condition	1.000	64.000	200.735	0.002	0.000	*0.964*

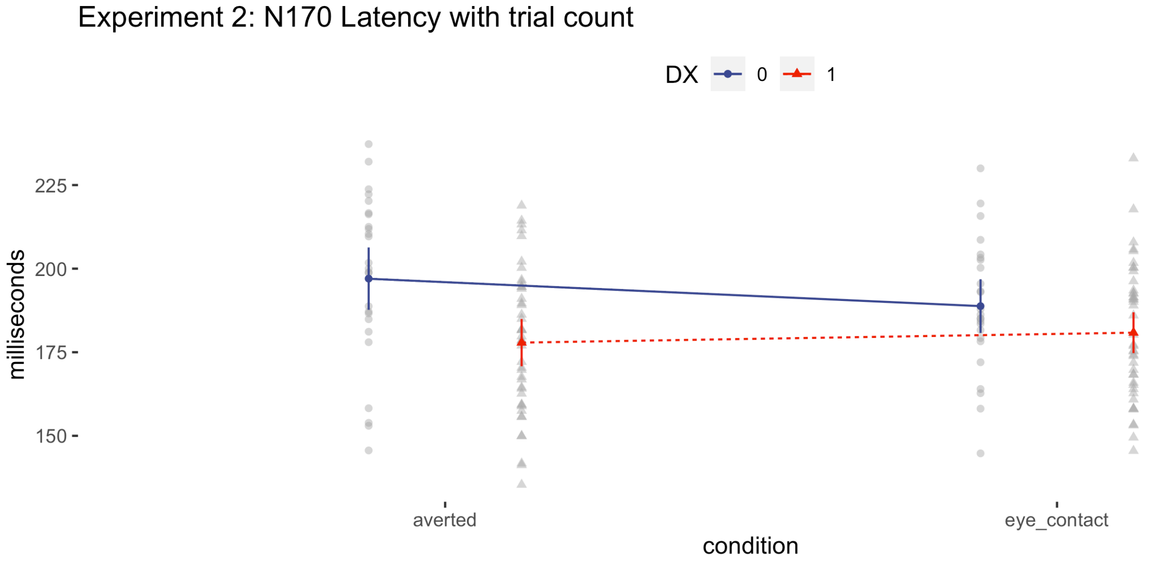

Table 10: *Experiment 2: N170 Amplitude with Trial Count*

	num Df	den Df	MSE	F	pes	Pr(>F)
DX	1.000	64.000	4.087	3.517	0.052	*0.065*
trialCount	1.000	64.000	4.087	0.034	0.001	*0.853*
DX:trialCount	1.000	64.000	4.087	1.238	0.019	*0.270*
condition	1.000	64.000	0.438	2.478	0.037	*0.120*
DX:condition	1.000	64.000	0.438	0.755	0.012	*0.388*
trialCount:condition	1.000	64.000	0.438	3.035	0.045	*0.086*
DX:trialCount:condition	1.000	64.000	0.438	3.971	0.058	*0.051*

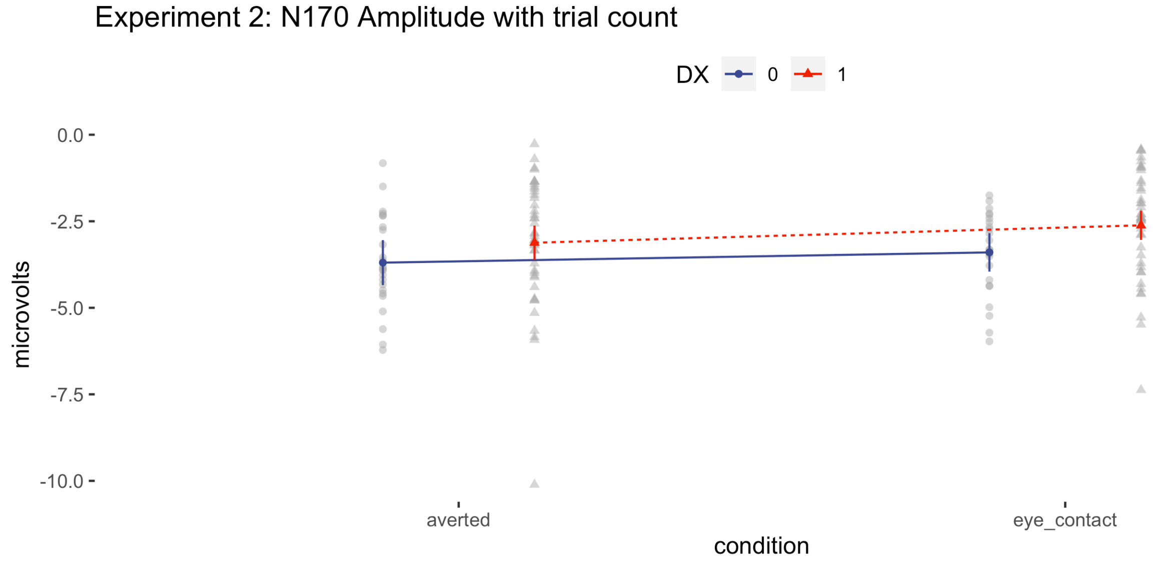

***Table 11**: Experiment 1: N170 Amplitude with Trial Count*

	num Df	den Df	MSE	F	pes	Pr(>F)
DX	1.000	65.000	7.478	0.969	0.015	*0.329*
trialCount	1.000	65.000	7.478	0.264	0.004	*0.609*
DX:trialCount	1.000	65.000	7.478	0.004	0.000	*0.951*
condition	1.000	65.000	1.008	26.883	0.293	*0.000*
DX:condition	1.000	65.000	1.008	5.961	0.084	*0.017*
trialCount:condition	1.000	65.000	1.008	2.536	0.038	*0.116*
DX:trialCount:condition	1.000	65.000	1.008	0.472	0.007	*0.495*

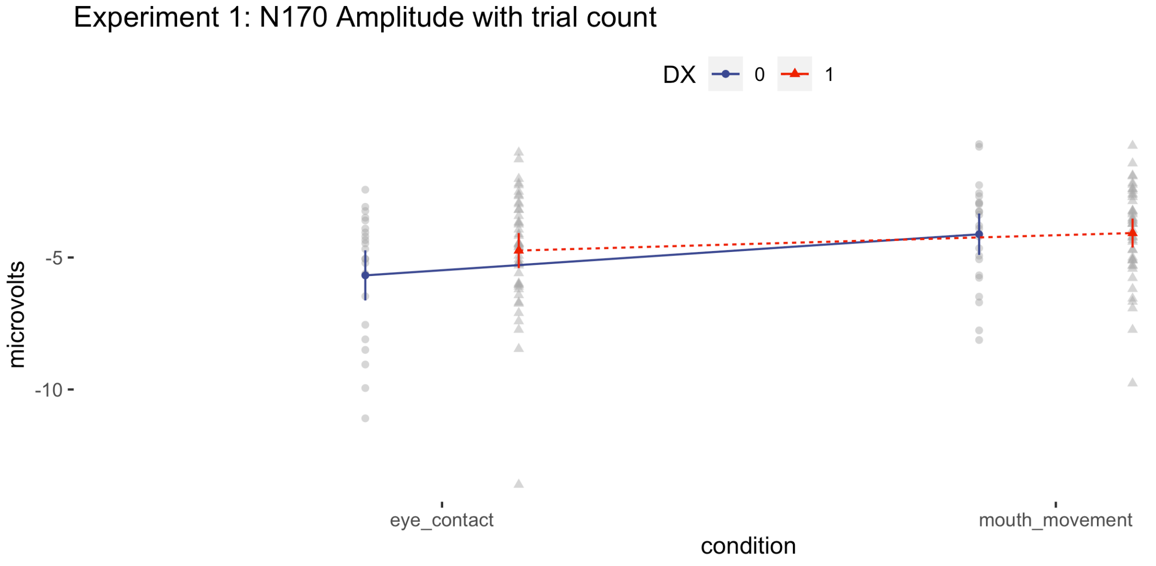

***Table 12**: Experiment 1: N170 Latency with Trial Count*

	num Df	den Df	MSE	F	pes	Pr(>F)
DX	1.000	65.000	576.764	0.365	0.006	*0.548*
trialCount	1.000	65.000	576.764	0.531	0.008	*0.469*
DX:trialCount	1.000	65.000	576.764	0.001	0.000	*0.982*
condition	1.000	65.000	124.854	1.767	0.026	*0.188*
DX:condition	1.000	65.000	124.854	1.391	0.021	*0.243*
trialCount:condition	1.000	65.000	124.854	0.021	0.000	*0.884*
DX:trialCount:condition	1.000	65.000	124.854	0.070	0.001	*0.792*

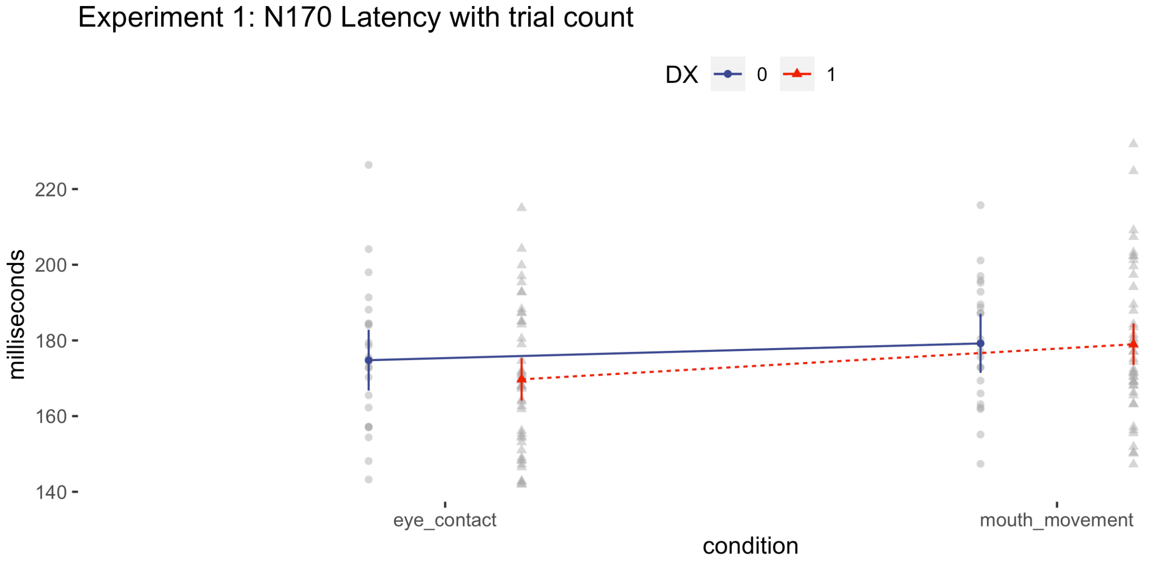

3. Analytic Models including IQ as a covariate

***Table 13**: Experiment 1: N170 Amplitude including IQ*

	num Df	den Df	MSE	F	pes	Pr(>F)
DX	1.000	28.000	8.151	0.624	0.022	*0.436*
IQ	39.000	28.000	8.151	0.824	0.535	*0.715*
condition	1.000	28.000	1.067	28.909	0.508	*0.000*
DX:condition	1.000	28.000	1.067	3.201	0.103	*0.084*
IQ:condition	39.000	28.000	1.067	0.937	0.566	*0.580*

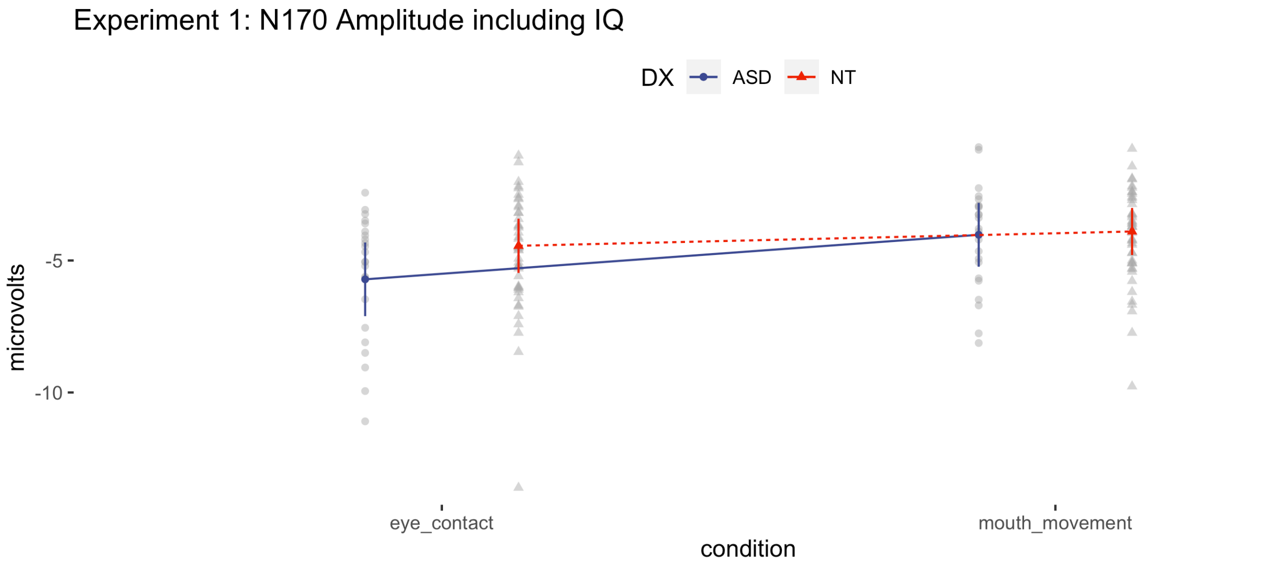

***Table 14**: Experiment 1: N170 Latency including IQ*

	num Df	den Df	MSE	F	pes	Pr(>F)
DX	1.000	28.000	664.917	1.034	0.036	*0.318*
IQ	39.000	28.000	664.917	0.757	0.513	*0.792*
condition	1.000	28.000	158.022	6.562	0.190	*0.016*
DX:condition	1.000	28.000	158.022	2.683	0.087	*0.113*
IQ:condition	39.000	28.000	158.022	0.607	0.458	*0.926*

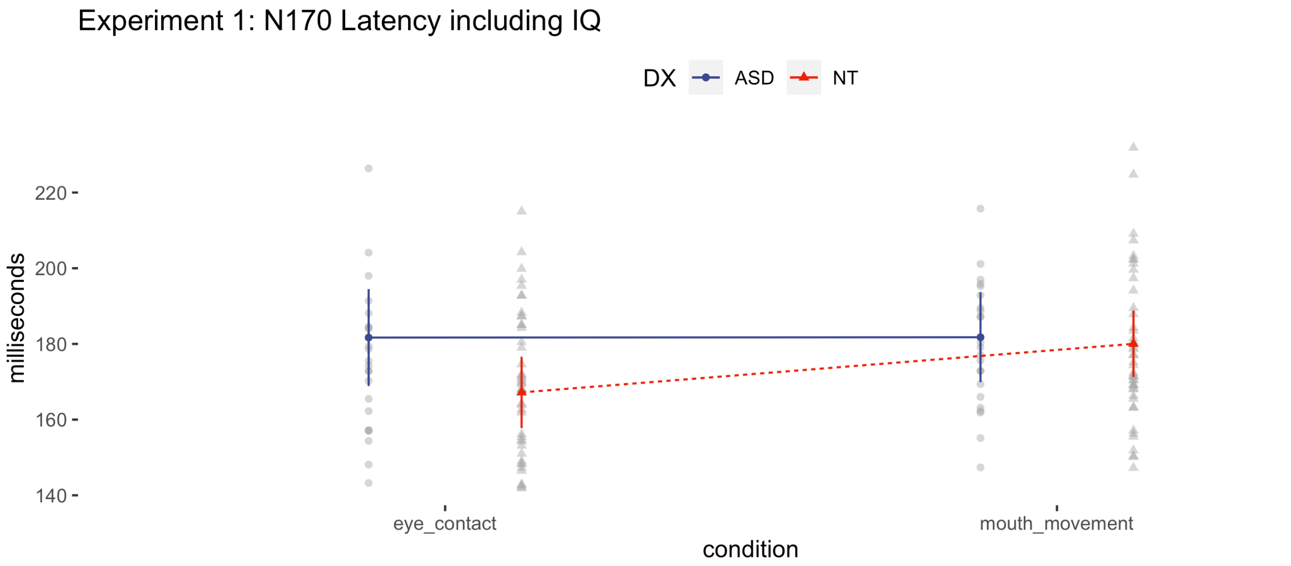

Table 15*: Experiment 1: P300 Amplitude including IQ*

	num Df	den Df	MSE	F	pes	Pr(>F)
DX	1.000	28.000	4.146	11.634	0.294	*0.002*
IQ	39.000	28.000	4.146	1.932	0.729	*0.036*
condition	1.000	28.000	0.762	7.097	0.202	*0.013*
DX:condition	1.000	28.000	0.762	4.823	0.147	*0.037*
IQ:condition	39.000	28.000	0.762	0.649	0.475	*0.895*

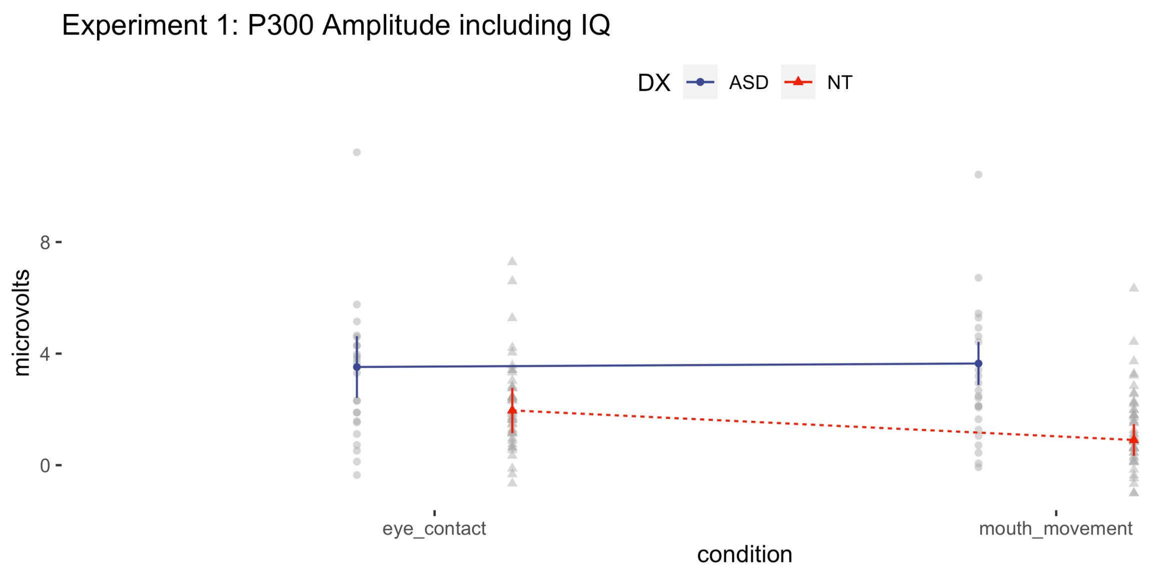

__Table 16__: Experiment 2: N170 Amplitude including IQ

	num Df	den Df	MSE	F	pes	Pr(>F)
DX	1.000	28.000	4.014	3.567	0.113	*0.069*
IQ	38.000	28.000	4.014	1.131	0.606	*0.371*
condition	1.000	28.000	0.401	10.188	0.267	*0.003*
DX:condition	1.000	28.000	0.401	0.000	0.000	*0.997*
IQ:condition	38.000	28.000	0.401	1.214	0.622	*0.299*

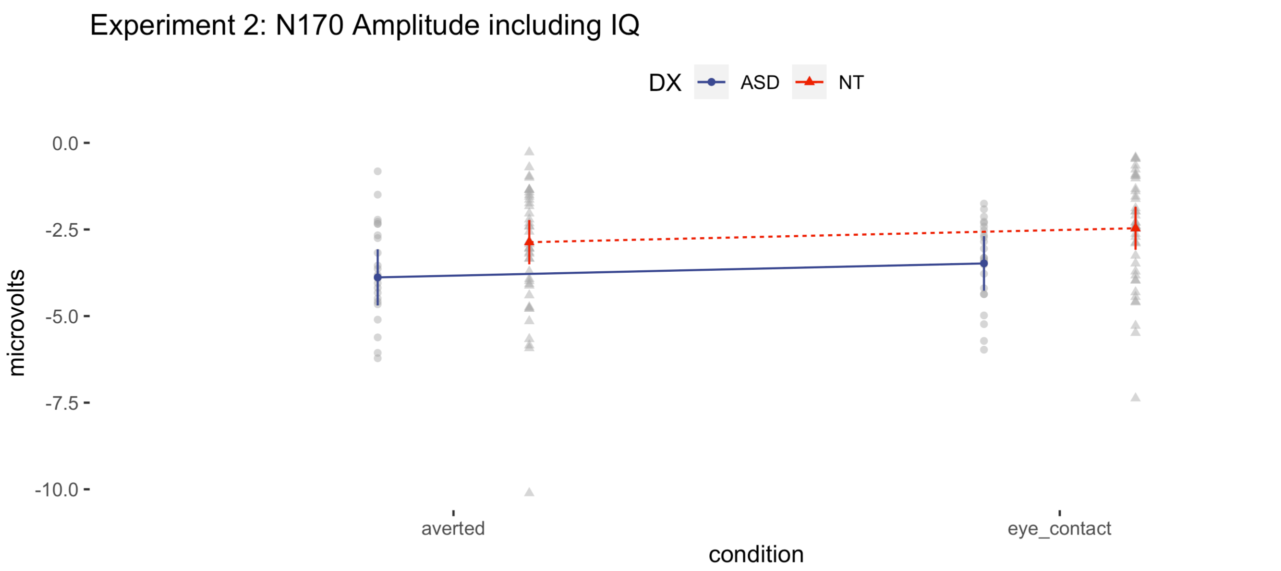

Table 17*: Experiment 2: N170 Latency including IQ*

	num Df	den Df	MSE	F	pes	Pr(>F)
DX	1.000	28.000	992.863	7.384	0.209	*0.011*
IQ	38.000	28.000	992.863	0.512	0.410	*0.973*
condition	1.000	28.000	224.035	0.985	0.034	*0.329*
DX:condition	1.000	28.000	224.035	7.254	0.206	*0.012*
IQ:condition	38.000	28.000	224.035	0.789	0.517	*0.755*

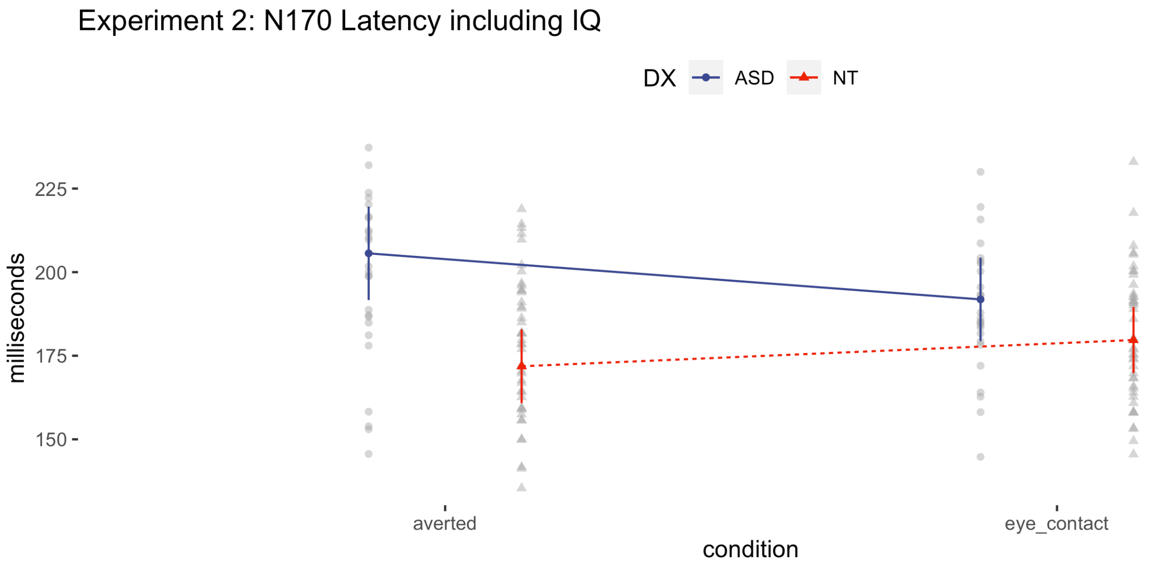

***Table 18**: Experiment 2: P300 Amplitude including IQ*

	num Df	den Df	MSE	F	pes	Pr(>F)
DX	1.000	28.000	2.524	0.630	0.022	*0.434*
IQ	38.000	28.000	2.524	1.001	0.576	*0.506*
condition	1.000	28.000	0.542	8.939	0.242	*0.006*
DX:condition	1.000	28.000	0.542	0.006	0.000	*0.939*
IQ:condition	38.000	28.000	0.542	1.885	0.719	*0.042*

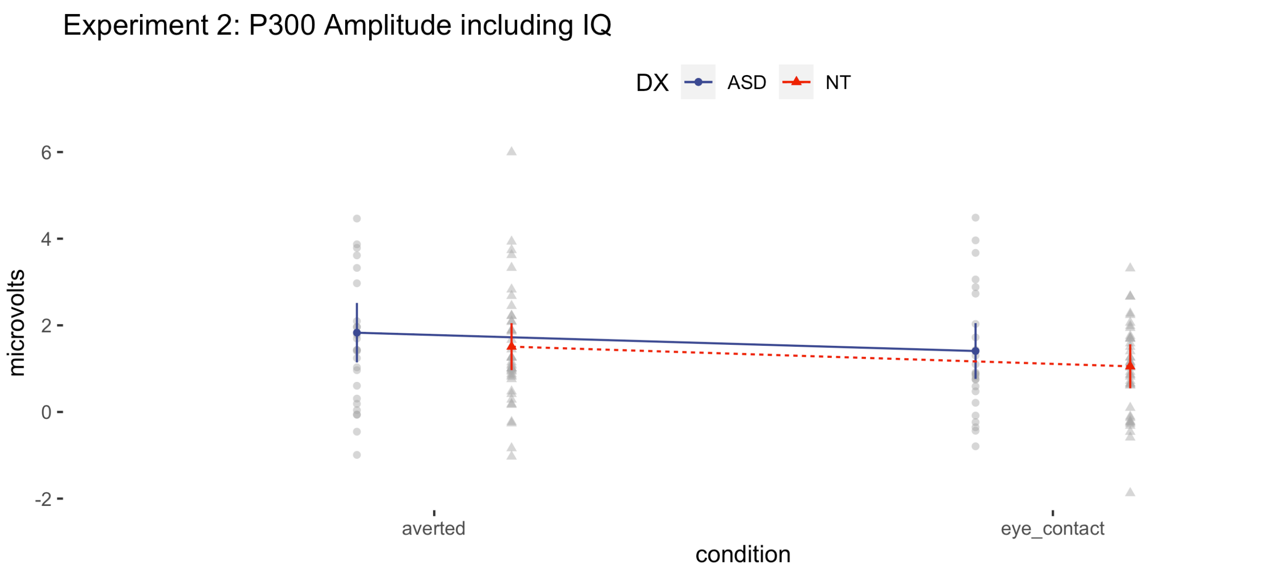

4. Means and standard deviations of dependent measures.

Table 19: *Means and SDs for all Dependent Variables*

DX	experimental_condition	component	mean	sd	N	experiment
ASD	eye_contact	N170 Amplitude	-5.599	2.358	23.000	*Experiment 1*
ASD	eye_contact	N170 Latency	175.190	19.244	23.000	*Experiment 1*
ASD	mouth_movement	N170 Amplitude	-4.123	1.971	23.000	*Experiment 1*
ASD	mouth_movement	N170 Latency	179.723	16.490	23.000	*Experiment 1*
NT	eye_contact	N170 Amplitude	-4.762	2.177	46.000	*Experiment 1*
NT	eye_contact	N170 Latency	169.571	18.589	46.000	*Experiment 1*
NT	mouth_movement	N170 Amplitude	-4.081	1.753	46.000	*Experiment 1*
NT	mouth_movement	N170 Latency	178.693	19.281	46.000	*Experiment 1*
ASD	eye_contact	P300	3.141	2.457	23.000	*Experiment 1*
ASD	mouth_movement	P300	3.061	2.432	23.000	*Experiment 1*
NT	eye_contact	P300	2.128	1.592	46.000	*Experiment 1*
NT	mouth_movement	P300	1.319	1.431	46.000	*Experiment 1*
ASD	averted gaze	N170 Amplitude	-3.681	1.356	25.000	*Experiment 2*
ASD	averted gaze	N170 Latency	197.175	25.250	25.000	*Experiment 2*
ASD	eye_contact	N170 Amplitude	-3.425	1.166	25.000	*Experiment 2*
ASD	eye_contact	N170 Latency	188.750	19.798	25.000	*Experiment 2*
NT	averted gaze	N170 Amplitude	-3.084	1.817	43.000	*Experiment 2*
NT	averted gaze	N170 Latency	177.869	21.297	43.000	*Experiment 2*
NT	eye_contact	N170 Amplitude	-2.590	1.538	43.000	*Experiment 2*
NT	eye_contact	N170 Latency	180.971	19.718	43.000	*Experiment 2*
ASD	averted gaze	P300	1.545	1.472	25.000	*Experiment 2*
ASD	eye_contact	P300	1.310	1.434	25.000	*Experiment 2*
NT	averted gaze	P300	1.463	1.331	43.000	*Experiment 2*
NT	eye_contact	P300	0.976	1.033	43.000	*Experiment 2*
ASD		N170 Amplitude Difference	-1.476	1.458	23.000	*Experiment 1*
ASD		N170 Latency Difference	-4.533	18.550	23.000	*Experiment 1*
NT		N170 Amplitude Difference	-0.682	1.422	46.000	*Experiment 1*
NT		N170 Latency Difference	-9.122	13.952	46.000	*Experiment 1*
ASD		P300 Amplitude Difference	0.080	1.078	23.000	*Experiment 1*
NT		P300 Amplitude Difference	0.809	1.113	46.000	*Experiment 1*
ASD		N170 Amplitude Difference	0.256	0.991	25.000	*Experiment 2*
ASD		N170 Latency Difference	-8.425	21.836	25.000	*Experiment 2*
NT		N170 Amplitude Difference	0.494	0.925	43.000	*Experiment 2*
NT		N170 Latency Difference	3.102	18.599	43.000	*Experiment 2*
ASD		P300 Amplitude Difference	-0.234	0.969	25.000	*Experiment 2*
NT		P300 Amplitude Difference	-0.487	1.426	43.000	*Experiment 2*
